# Supplementary material for: A Humanized Yeast Phenomic Model of Deoxycytidine Kinase to Predict Genetic Buffering of Nucleoside Analog Cytotoxicity
Source: Genes (Basel). 2019 Sep 30;10(10):770. doi: 10.3390/genes10100770 (PMC6826991; doi:10.3390/genes10100770)
Supplement: Supplementary file 1 [file genes-10-00770-s001.zip › Additional_Files/Additional_File7_GOTermHeatmaps.zip!/Additional_File7_GOTermHeatmaps/four-way_junction_helicase_activity.pdf]

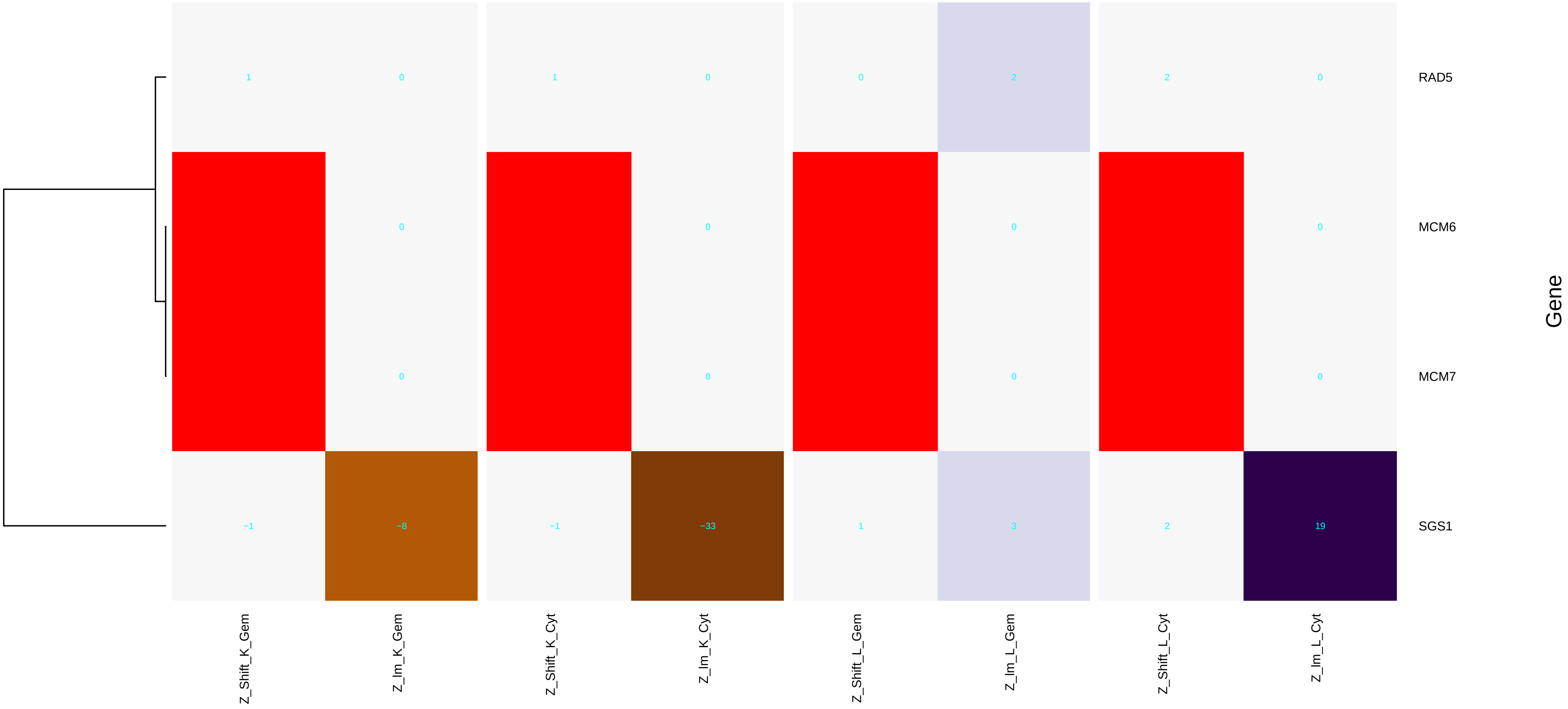

RAD5
MCM6
MCM7
SGS1
Gene
Z_Shift_K_Gem
Z_Im_K_Gem
Z_Shift_K_Cyt
Z_Im_K_Cyt
Z_Shift_L_Gem
Z_Im_L_Gem
Z_Shift_L_Cyt
Z_Im_L_Cyt
1
0
1
0
0
2
2
0
0
0
0
0
0
0
0
0
−1
−8
−1
−33
1
3
2
19
